# The Ubuntu Way: Ensuring Ethical AI Integration in Health Research

**DOI:** 10.12688/wellcomeopenres.23021.1

**Published:** 2024-10-28

**Authors:** Brenda Odero, David Nderitu, Gabrielle Samuel

**Affiliations:** 1 Strathmore University, Nairobi, Nairobi County, Kenya; 2 School of Law, University of KwaZulu-Natal - Pietermaritzburg Campus, Pietermaritzburg, KwaZulu-Natal, South Africa; 3 Egerton University, Njoro, Nakuru County, Kenya; 4 Department of Global Health and Social Medicine, Bush House, North East Wing, Strand, King's College London, London, England, UK

**Keywords:** Ubuntu, Health Research, Ethics, Artificial Intelligence (AI), Africa

## Abstract

The integration of artificial intelligence (AI) in health research has grown rapidly, particularly in African nations, which have also been developing data protection laws and AI strategies. However, the ethical frameworks governing AI use in health research are often based on Western philosophies, focusing on individualism, and may not fully address the unique challenges and cultural contexts of African communities. This paper advocates for the incorporation of African philosophies, specifically *Ubuntu,* into AI health research ethics frameworks to better align with African values and contexts.

This study explores the concept of *Ubuntu,* a philosophy that emphasises communalism, interconnectedness, and collective well-being, and its application to AI health research ethics. By analysing existing global AI ethics frameworks and contrasting them with the *Ubuntu* philosophy, a new ethics framework is proposed that integrates these perspectives. The framework is designed to address ethical challenges at individual, community, national, and environmental levels, with a particular focus on the African context.

The proposed framework highlights four key principles derived from *Ubuntu*: communalism and openness, harmony and support, research prioritisation and community empowerment, and community-oriented decision-making. These principles are aligned with global ethical standards such as justice, beneficence, transparency, and accountability but are adapted to reflect the communal and relational values inherent in *Ubuntu*. The framework aims to ensure that AI-driven health research benefits communities equitably, respects local contexts and promotes long-term sustainability.

Integrating *Ubuntu* into AI health research ethics can address the limitations of current frameworks that emphasise individualism. This approach not only aligns with African values but also offers a model that could be applied more broadly to enhance the ethical governance of AI in health research worldwide. By prioritising communal well-being, inclusivity, and environmental stewardship, the proposed framework has the potential to foster more responsible and contextually relevant AI health research practices in Africa.

## Introduction

The 2023 Government AI Readiness Index by Oxford Insights revealed that an ever-increasing number of African nations, such as Egypt, Mauritius, Senegal, Benin, and Rwanda are developing national AI strategies for effective AI adoption ( Oxford Insights, 2023). This index is assessed against three key pillars: Government (regulation, ethical risks, and AI governance), Technology (availability and usage of AI tools), and Data and Infrastructure (quality and unbiased data representing citizens). Additionally, Sub-Saharan African countries, including Kenya, Uganda, South Africa, and Zambia, have now enacted data protection laws and regulatory frameworks for secure data handling, including for the use of AI, emphasising consent in using citizens' data ( Bosire, 2022; Onuoha, 2019). Most recently, at least 36 out of 54 nations have implemented formal data protection laws, providing a solid base for developing comprehensive AI regulations ( Hogan Lovells, 2023).

While national regulations and guidelines are in place for some African countries, these jurisdictions have paid less attention to how these policies specifically relate to the use of AI in health research. This is troublesome given that AI is already being used by researchers in some of these countries for aiding with health diagnosis and researching health-related topics (e.g. by using electronic medical records, public health surveillance, prediction of outbreaks, and health policy and planning). It is also troublesome because poor regulatory frameworks increase the chances of ethics dumping - a practice already widespread in Africa - as Global North researchers may look to train algorithms on health data sets in a way that bypasses regulations in their own countries ( Chatfield *et al.*, 2021). 

Some authors have developed proposed research ethics frameworks to help address the potential harms that may arise through AI health research in Africa, including the issue of ethics dumping ( Odero & Nderitu, 2022). These frameworks typically originate from a Global North perspective and along with many other frameworks in the AI innovation space, have used a Global North philosophy to contextualise harms, i.e., one that focuses on ideals of individualism or the Western reductionist and positivist conception of harm ( Shaw & Barrett, 2006). Notions of individualism in research ethics have served the international community well over the past half a century, ensuring that biomedical and other research is conducted to protect research participants from harm and promote social value through cost-benefit assessments ( Chattopadhyay & De Vries, 2008; Wilkinson, 2004). However, the research arena has changed considerably over the last few decades: research methods have expanded to include digital and AI research, and globally, concerns about social and environmental justice, sustainability, and climate change have come to the fore. Attention has focused on the limitations of current research ethics models to address these changes. Alongside this, a growing movement of African scholars has argued that AI ethics frameworks should be guided by more traditional African philosophies. Gaffley *et al.* (2022) suggest that the development of ethical principles and guidelines governing the usage of AI technologies that are formulated based on African values and standards - in particular, those which are more relational in approach rather than based on individualism ( Ewuoso, 2021) - have the potential to generate responsible AI perspectives that can address the specific AI-related challenges experienced on the Continent. To not incorporate these perspectives, argue these authors, would be a disservice to African countries.

In this paper, we align with such scholars, stressing the importance of embedding African philosophies within African countries’ AI health research ethics frameworks. At the same time, we do not reject research ethics frameworks that have come before. Rather, we emphasise the importance of taking what has already worked well at the international research ethics level and integrating this with indigenous African worldviews. We have developed a framework for AI health research ethics that integrates existing relevant global philosophies within Indigenous African worldviews to establish a relevant and contextual ethics framework relevant to the African context. Furthermore, our approach to AI health research ethics need not stop at the African continent but can address some of the limitations imposed by individualistic approaches that we see in research ethics frameworks at the international level.

In the remainder of this paper, we develop our claims and present our framework. We do this in five sections. First, we briefly introduce the topic of AI health research ethics, particularly the suite of artificial intelligence (AI) methodologies currently used in health research both internationally and within Africa, and the ethical issues associated with their use. Second, we introduce the well-established indigenous African concept of *Ubuntu*, which we use as our framing for the ethical framework; we illustrate the relational aspects of this concept and compare it to the more individualistic worldview predominant in the Global North. Third, we describe how a relational approach that draws on *Ubuntu* philosophy provides a useful framing to support the use of AI in health research, allowing for ethics to be considered between individuals and communities, as well as the environment. Fourth, we introduce our framework. Finally, we discuss some limitations of our approach.

## Artificial Intelligence (AI)

Current AI approaches encompass a wide range of self-learning data analysis techniques trained on large datasets. Machine learning is probably the most common AI technique used in health research and can be defined as a *‘set of methods that can detect patterns in data automatically to predict data trends or for decision-making under uncertain conditions’* ( Ho *et al.*, 2019). Machine learning has various sub-categories, including deep learning, which learns unsupervised from unstructured data; neural networks - algorithms that are most used for image analysis; and natural language processing, which focuses on analysing human language. Generative AI, such as ChatGPT, Dall-E and Bard, and virtual assistants like Alexa and Siri, are recent iterations of AI that have received perhaps the most public, policy and media interest. Other AI techniques include embodied AI robotics.

AI technologies have been applied in a range of research areas, settings, and domains. The health sector has been a slow adopter of AI ( Wiljer *et al.*, 2021), but the massive increase in health-related datasets has led to excitement at the prospect of this technology delivering real-world impacts to health and has seen developments advancing at a rapid pace. Vast swaths of clinical and associated data, such as electronic health records, imaging data, ‘omics data (proteomics, genomics, metabolomics etc.), social media data, and/or other passive data (location, sleep hours, tracker information) all offer potential datasets for AI testing and training. The analysis of these datasets promises to improve the diagnosis of certain cancers and the detection of genetic disorders, improve the prediction of responses to certain medications, and predict various health and mental health states ( Birk & Samuel, 2020; Blasimme & Vayena, 2019; Gurovich *et al.*, 2019; Loh, 2018; Tran *et al.*, 2019). AI algorithms are also being used in drug discovery and clinical trials, and for advancement in public and preventative health, including epidemic, pandemic, and disease outbreak control ( Bengtsson *et al.*, 2015; Chen & Asch, 2017; Harris *et al.*, 2017; Naghavi *et al.*, 2010; Subbiah, 2023).

In the African context, the use of AI in health research has also increased at a rapid rate ( Townsend *et al.*, 2023). In Nigeria, a startup called Ubenwa is utilising signal processing and machine learning to enhance the diagnosis of birth asphyxia in low-resource settings ( Onu *et al.*, 2019). In Kenya, chatbots are currently employed to offer healthcare services to individuals without the need for in-person doctor visits ( Francesc *et al.*, 2019). In Zambia, Bellemo *et al.* (2019) demonstrated promising results in diagnosing diabetic retinopathy using AI, showing comparable performance to human assessments, and detecting referable diabetic retinopathy effectively ( Bellemo *et al.*, 2019). AI has also made a positive impact on the pharmaceutical industry in Nigeria. Five high school girls developed an app based on MIT open-source software to identify counterfeit drugs in the country, receiving recognition and winning a contest in Silicon Valley in 2018 ( Akande, 2018). Furthermore, the Delft Institute's CAD4TB software has been used in pilot studies in Tanzania and Zambia to aid in the computer-aided diagnosis of pulmonary tuberculosis from chest radiographs, with results that are comparable to those of human experts ( Breuninger *et al.*, 2014; Melendez *et al.*, 2017).

## Health Research Ethics and AI

Ethical concerns associated with the use of AI in health research can be categorised into four levels. First, at the *individual* level, concerns have been raised about the protection, rights, safety, and welfare of *data participants*, including the need to provide respect and protect their privacy in terms of personal data access control, protection of sensitive data and its use ( Blasimme & Vayena, 2019; Cath *et al.*, 2018; Dignum, 2018; Vayena *et al.*, 2018; Vollmer *et al.*, 2018). Secondly, ensuring the protection of *communities* who are affected by research from harms that may be caused as a result of discrimination associated with algorithmic bias through considerations of fairness and justice, as well as ensuring equity of access to any health care interventions that result from the AI research ( Blasimme & Vayena, 2019; Cath *et al.*, 2018; Dignum, 2018; Owoyemi *et al.*, 2020; Vayena *et al.*, 2018; Vollmer *et al.*, 2018). Such discrimination has already been reported outside of the health arena. For example, Adams (2022) points to how the use of facial recognition technologies in African cities, from Kampala to Johannesburg, have not been trained on local facial data which has led to the misreading of faces and other forms of human rights limitations, especially in settings where social ills have been perennial. In the health arena, but outside of Africa, Obermeyer *et al.* (2019) showed how one AI system, which was used in healthcare to decide which patients with serious health issues needed long-term care, favoured white patients with the same medical problems. Such challenges are likely to also emerge in the African context because much health research in Africa is underpinned by software developed outside of the continent using datasets with a low prominence of African data subjects. Third, at *the national level,* concerns have been raised about the lack of value of health research to people living in Africa. Otaigbe (2022) points to the often, short-term pilot health research that is implemented in the deployment of AI for infectious diseases in African communities, which he explains, often operates in silos, with little integration into African healthcare systems. This reduces long-term sustainability, leading to a waste of both valuable resources and time.

Finally, there is a need to protect the *environment* affected by the manufacturing, use, and waste disposal of equipment, tools, and technologies associated with AI-related health research. This includes the large amounts of energy consumed to power and cool the data centres that store and process data (often in the form of fossil fuels, though increasingly this is changing to renewable energy). The excess consumption of electric power used by data centres can redirect electricity away from domestic use, especially in low-resource settings such as sub-Saharan Africa where electricity generation is still insufficient for all domestic use ( Owoyemi *et al.*, 2020). Environmental impacts also include the depletion of natural mineral resources in Sub-Saharan Africa - countries which have become geared towards developing technology hardware - as well as the environmental harms associated with the dumping of digital hardware waste in African countries. Such harms are compounded by the lack of regulation governing many of these processes and practices meaning that they are implicated in a range of extractivist and exploitative practices ( Lebbie *et al.*, 2021). For example, many communities in African countries make a living by recycling minerals extracted from digital components in digital hardware dump sites (e-waste dump sites) by using hazardous chemicals/acid baths that not only have a detrimental health impact on themselves and their families ( Orisakwe *et al.*, 2019) but also on the environment.

Many of the ethical considerations associated with the use of AI health research have been articulated more widely in a range of international and national guidelines, recommendations, statements, and documents ( Farhud & Zokaei, 2021; Kargl *et al.*, 2022). Presently, more than 600 policy recommendations, guidelines, and strategy reports on AI have been issued by notable intergovernmental organisations, professional associations, committees at the national level, public institutions, non-governmental entities, and for-profit companies in the private sector ( OECD, n.d.), and to date at least 84 AI Ethics initiatives have published reports describing high-level ethical principles, values, or other abstract requirements for AI development and deployment ( Jobin *et al.*, 2019). These include the need to ensure public and stakeholder trust in AI systems, the need for accountability of the technology (including questions of responsibility), the need to ensure transparency and explainability of the algorithms, as well as the need to ensure the principles of justice, fairness, non-maleficence, privacy, beneficence, freedom, autonomy, sustainability, dignity, and unity are upheld ( Algorithm Watch, 2020). However, these guidelines have been predominantly developed in a non-African context and have prioritised individual and community issues associated with AI research and use, with much less attention given to concerns towards the national level and environmental safety concerns ( Maes & Preston-Whyte, 2022).

## What is *Ubuntu?*

The concept of *Ubuntu* is found in nearly all African Bantu languages. It shares its roots with the word ‘bantu’ meaning ‘people’ is also known as African humanism and as an ideology it is manifested beyond the Bantu communities. *Ubuntu* immediately denotes the essential need for community and interconnectedness with people. The best representation of *Ubuntu* is in both the Xhosa and Zulu proverb: ’umuntu ngumuntu ngabantu’ which means a person is a person through other persons. This is reflected in the Kiswahili saying, ‘Mtu ni watu’ which is loosely translated as ‘a person is people’ and has the connotation that a human being finds identity by being in the community. A great deal of African cultural proverbs dwell on the importance of togetherness in fulfilling human existence. *Ubuntu* or other closely related words are found in different African countries and cultures. In Rwanda and Burundi, it means ‘human generosity’. The Swahili word ‘ *utu*’ means every action should benefit humanity.

John S. Mbiti summarised the conceptualization of *Ubuntu* philosophy in a statement: ‘I am because we are, and since we are therefore, I am’ ( Mbiti, 1975). This simply means that an individual cannot exist without the others. Therefore, there is no person but the community; there is no “I” but “we”. In this sense, it may be translated to mean that if a person does not relate well with others, then they are not a person ( Metz, 2010). In essence, *Ubuntu* seeks to promote the dignity and integrity of human beings by promoting communal good and propagating common humanity by demonstrating the interconnectedness of all human beings, irrespective of where they live ( Molefe & Magam, 2019).

Despite the growing influence of individualism in Africa ^1^, *Ubuntu* still reigns in most contexts. *Ubuntu* is culturally inculcated from childhood; socialisation in most African cultures is conducted in groups of age mates or contemporaries called age sets (the Agikuyu of Kenya refers to this as *riika*). This inculcates a sense of belonging, relatedness, and obligation to a more extensive set of other individuals ( Ekore & Lanre-Abass, 2016). Ordinarily, children would gather for day-to-day communal chores like grazing and fetching water and in significant stages of life like the rites of passage which are often rituals for age sets.

It is important to note that the concept of communalism is not exclusive to Africa. There is evidence of ‘ *Ubuntu’* in many other parts of the world including the Global North and there are cases where communitarianism would be inevitable in Western cultures ( Nderitu, 2020). It is also important to note that while *Ubuntu* remains a vital and active part of African life, this does not mean that individual choice is not important in African countries; there are instances where individual choices and decisions would be more valuable than community ones in Africa. However, by and large, *Ubuntu* remains a significant ideology in Africa which can be used to inspire global values.

## Relevance of *Ubuntu* in international health research and integration of AI

In the research setting, *Ubuntu* would be translated to the ethical obligation to promote research participant welfare and community interest. While this has similarities with more international framings of research ethics based on Western philosophies, Western approaches have insisted on the centrality of autonomy because individuals are viewed as having moral standing *separate* from other individuals, such that they can make moral rational choices independently of others. Furthermore, ethical concepts in the West frequently emphasise reasoning and logic, rather than feelings and intuition, resulting in ethical conclusions that are excessively theoretical and lack a direct correlation with people's real-life experiences ( Amugongo *et al.*, 2023). This Western perception can be traced back to the classical Greek period in antiquity to the modern and contemporary Western thinkers, with the basic understanding of a human person being a rational animal or an individual substance of rational nature, or even an individual entity composed of a rational soul informing a material body. This is evident in the description by Aristotle, Boethius and Thomas Aquinas ( Nderitu, 2020). But in Africa, the interpretation of autonomy has often been about respect for persons *because that person -rather than being an individual of a rational nature - is part of a community.* If one harms or exploits another person, or if a research engagement harms the community or the environment within which the community lives then the values of *Ubuntu* are strained. *Ubuntu* would remind researchers to view individual research participants as being integral parts of the community and sometimes making decisions as individuals would need the blessing of the community or its representatives. It would also mean that the interpretation of key research concepts like benefit, harm, and justice are approached from the community rather than the researcher’s perception. Arguments from scholars emphasise the relevance of *Ubuntu.* For example, Mugumbate and Nyanguru (2013) assert that *Ubuntu* introduces a human aspect to all facets of life, a quality they believe Western civilization did not achieve. The importance of this human element in intricate ethical choices is particularly evident in social work. Mabvurira (2020) contends that the ethical framework of *Ubuntu* not only provides a solid foundation for ethical decision-making among African people but also aligns well with many principles and ethical theories in research ethics.

There are growing calls to integrate *Ubuntu* into research practices both in Africa and more broadly and for the decoloniality of African research away from Global North assumptions ( Chaurey, 2020; Seehawer, 2018). In AI health research, integrating the *Ubuntu* philosophy can enhance existing research ethics frameworks by promoting mutual respect, community involvement, equity, compassion, and socially valuable outcomes at different levels of ethical deliberation. First, health research involving AI creates challenges and ethical concerns related to receiving consent that *Ubuntu*-inspired community engagement can help with because communitarian African contexts require complementary research practice frameworks like community and/or group consent, a gap which can be filled by this model ( Moodley & Beyer, 2019). Second, *Ubuntu*, and its emphasis on community, can be harnessed to emphasise inclusivity in AI development. Furthermore, its focus on relationality can guide decisions associated with the development of algorithms in a way that will not discriminate unjustly ( Ferlito & De Proost, 2023; Gwagwa *et al.*, 2022). Here, *Ubuntu* goes beyond the common understanding of justice as fairness—and especially distributive justice which dominates research ethics because of its three outstanding principles of contribution, need and equality—offering the values of communal sharing of resources, mutual responsibility and welfare. Finally, and importantly, *Ubuntu* allows consideration of the environmental implications of AI health research—something often lacking in international frameworks—because it promotes a sense of interconnectedness and collective responsibility towards the environment: in essence, in the spirit of *Ubuntu,* we need to consider the earth as a common good ( Oruka & Juma, 1994) such that it is the responsibility of every human person to preserve the earth through activities that promote the environment’s conservation for this common good. In other words, *Ubuntu* is by and large a campaigner for human flourishing by ensuring egalitarian responsibility from each person to sustain the environment for the present and future generations.

## Community Good as the Centre of AI-Driven Healthcare Research Derived from *Ubuntu*

In the context of AI health research, the *Ubuntu* philosophy can offer significant ethical guidance, emphasising community-oriented values over individualistic approaches. To develop this guidance, we present four principles/practices to represent the key norms associated with the concept of *Ubuntu.* We have aligned each of these principles/practices with those typically already formulated in international frameworks to bridge the relationship between *Ubuntu* and wider Global frameworks. Of note, our choice of principles/practices terminology comes from the acknowledgement that the relationship between principles and practices is sometimes blurry, with some principles also actions in themselves and therefore, there may not be clear distinctions between such concepts in our discussions. We propose our principles/practices are vital to guide and shape the kind of ethical research practices that would be essential for AI health-related research in a context dominated by communalistic worldviews.

1. **Communalism and openness:** *Ubuntu* promotes the idea that an individual's well-being is closely tied to the well-being of their community. This contrasts with more Western individualistic approaches and can guide AI development to consider the impacts on entire communities rather than focusing solely on individual outcomes. In AI health research, community can be translated as community engagement, collaborative partnerships between stakeholders, ongoing respect for the communities being researched, and respect for their environment and those affected by those environments. This communal approach can lead to the design of AI systems that support community health initiatives and improve overall community health services, rather than focusing solely on personalised medicine ( Van Norren, 2022).
**Includes other ethical principles/practices of:**
**Justice**: Communalism emphasises fair treatment and equity during research, ensuring that the community being researched has access to health resources and services. This aligns with the ethical principle of justice, which advocates for fairness and equality in distributing benefits and risks. This aligns with the basic tenets of justice including contribution, need/equity and equality in selecting research communities and distributing the benefits of research.
**Beneficence**: By promoting the welfare of the entire community, communalism aligns with beneficence, seeking the greatest good for the greatest number and prioritising communal well-being.
**Transparency and Explainability**: These principles, emphasise the importance of clear, understandable AI processes, and align well with *Ubuntu*’s focus on openness and communal understanding. Transparent AI systems ensure that decisions made by AI are understandable to all interest holders.
2. **Harmony and Support:** The practice of harmony in *Ubuntu* underscores mutual support within communities. For AI health research, this could mean developing systems that facilitate community-based health initiatives, such as AI-driven platforms that enhance communal health services or tools that support community health workers. The goal would be to ensure that these technologies foster a sense of inclusion and support, guaranteeing that no community member is left behind in accessing health services ( Van Norren, 2022). For example, Vishwanatha *et al.* (2023), determined that holding a nationwide listening session led to the enhancement of AI/ML data, infrastructure, and training by prioritising community input, thereby enabling interest holders to drive meaningful change when it came to health research that integrates AI.
**Includes other ethical principles/practices of:**
**Fairness and Non-Discrimination**: This principle which is an element of justice ensures that AI systems do not create or perpetuate inequities or unfair biases, aligning with *Ubuntu’s* emphasis on communal harmony and the equitable treatment of all community members. AI systems must be designed to serve diverse communities fairly, a core component of *Ubuntu’s* ethical considerations and prioritization of community welfare ( UNESCO, 2022).
**Responsibility and Accountability**: Accountability in AI requires that creators and operators of AI systems are responsible for the outcomes of these systems. In an *Ubuntu*-influenced framework, this could translate into community-level oversight and accountability, ensuring that AI systems benefit the community and do not harm societal cohesion ( UNESCO, 2022). For example, this includes preventing the misuse of data in ways that do not benefit the community. Working in solidarity with the community can ensure that any unforeseen event from AI health research is explained and accounted for acceptably.
3. **Research prioritisation and community empowerment:** In line with *Ubuntu*, AI health research should only be conducted if there is infrastructure in place to ensure that resulting technologies will be allocated equitably, considering the needs of the most vulnerable and marginalised. Prioritised research must ensure long-term infrastructures are established for equal and inclusive access and mitigate environmental impacts to protect communities affected by the manufacture, use, and disposal of digital technologies. This approach ensures that AI technologies are accessible to underserved communities and work to diminish, rather than exacerbate, existing health disparities ( UNESCO, 2022).
**Includes other ethical principles/practices of:**
**Justice**: Prioritising the needs of the most vulnerable aligns with the principle of justice, focusing on equity and the fair distribution of AI benefits to address health disparities.
**Non-maleficence**: By considering the ethical implications of AI deployment, such as avoiding harm to marginalised groups, this practice upholds the principle of non-maleficence.
**Sustainability**: This principle demands that AI technologies are assessed for their long-term impact on the environment and society. In *Ubuntu*, sustainability would emphasise the survival and thriving of the entire community, ensuring that AI development supports long-term communal goals, including environmental stewardship which is part of community empowerment and reparative justice.
4. **Community-oriented decision-making:** Consistent with *Ubuntu*'s emphasis on communal decision-making, involving community stakeholders in the development and implementation of AI health research can ensure that these technologies are aligned with community values and needs. I. For instance, obtaining consent for research often involves not only individual agreement but also communal approval and the endorsement of local gatekeepers. This might include participatory design processes where community members have a say in how AI tools are developed and implemented in their healthcare systems, ensuring that the outcomes are beneficial and accepted by all members of the community ( UNESCO, 2022). The outright principle that emerges from this aspect is **community engagement** throughout the life cycle of AI. The relational aspect of consent is a significant factor to consider, as it highlights that decision-making is not always the result of rational, autonomous choices by individuals. Instead, consent is often shaped by the relationships and connections individuals have with others, whether within their immediate community, their family, or a broader social network. This perspective underscores the importance of understanding consent as a process influenced by social and relational contexts, rather than as a purely individualistic or isolated act.
**Includes other ethical principles/practices of:**
**Safety and Security**: These principles focus on preventing harm from AI systems, which is crucial in an *Ubuntu* framework that prioritises the well-being and safety of the community. Ensuring that AI systems are safe and secure prevents them from undermining community trust and cohesion.

## Limitations of the *Ubuntu Philosophy* in AI Health Research

Currently, there is a growing enthusiasm for Ubuntu as an alternative ethical framework to Western principle-based approaches. However, as is often the case with new concepts, we must be cautious to avoid its misappropriation, work to ethically analyse it as a concept to better understand its moral work, and then acknowledge its inherent limitations. De Vries (2024) points to two problems regarding the reference to Ubuntu in scholarship: first is the impression that African philosophy, and ideas around Ubuntu, are settled, stable and uncontested, and second is the feminist concern that Ubuntu could be promoting conservative patriarchy that continues to silence the voices and perspectives of others. Our concern, just like De Vries’, is that scholars’ reference to Ubuntu without critical analysis may lead to portraying the worldview as absolute, which undermines the purpose that it may want to serve. Our scrutiny of the concept is on what it could mean in practical application in some African cultural contexts. For example, principles provide scaffolding for ethical frameworks, but it is well-documented that their abstract nature provides little guidance to actors making decisions at the level of practice. This is also true of *Ubuntu*, for which the biggest limitation is its lack of clarity around the notion of ‘community’, and whether ‘the community’ should be local, national, or international. Cosmopolitan approaches to ethics (where issues of moral worth extend outside nations) are now deemed most moral for a globalised world, but historically, *Ubuntu* is a local concept, where the concept of a human person is often limited to a given ethnic community or related communities, and persons from other ethnicities are regarded as less human hence the derogatory names given to "outsiders" ( Nderitu, 2020). While there may have been ways of handling friendly outsiders, there is a possibility that *Ubuntu* may have little to extend to the globe and may not be about global issues. This should not deter us because the practical local value of *Ubuntu* in enhancing egalitarian goods has often been linked to the potential of informing best practices that espouse the ideals of universally accepted principles like utilitarianism. It is crucial that we clearly define Ubuntu’s theoretical and practical applications to ensure that it can help our ethical decision-making rather than doing more harm than good.

## Ethics and consent

Ethical approval and consent were not required.

## Data Availability

No data are associated with this article.

## Footnotes

^1^Individualism understood to refer to favouring of individual freedom and autonomy has increased especially among the urban dwellers in most African countries supposedly due to exposure to the Western culture, capitalism and modernisation.
