# Supplementary material for: The Ubuntu Way: Ensuring Ethical AI Integration in Health Research
Source: Wellcome Open Res. 2024 Oct 28;9:625. [Version 1] doi: 10.12688/wellcomeopenres.23021.1 (PMC11599802; doi:10.12688/wellcomeopenres.23021.1)
Supplement: Peer review history

# Reviewer report: Reviewer response for version 1

**DOI:** 10.21956/wellcomeopenres.25352.r108620

**Published:** 2024-11-26

**Authors:** Balaram Yadav Kasula

**Affiliations:** 1 University of the Cumberlands, Williamsburg, Kentucky, USA

The paper presents a compelling argument for integrating the African philosophy of Ubuntu into AI health research ethics frameworks to better align with the communal values and cultural contexts of African communities. By contrasting existing global AI ethics, often grounded in Western individualism, with Ubuntu's principles of interconnectedness, collective well-being, and community empowerment, the study proposes a novel ethics framework tailored to African realities. This framework incorporates four key principles: communalism and openness, harmony and support, research prioritization, and community-oriented decision-making, aligning with global standards like justice and transparency but adapted for communal values. The approach addresses ethical challenges at multiple levels—individual, community, national, and environmental—ensuring that AI health research benefits are equitably distributed, respectful of local contexts, and sustainable. This integration not only enhances the ethical governance of AI in African health research but also offers a scalable model for global application, promoting inclusivity and long-term well-being in AI-driven health initiatives.

Is the review written in accessible language?

Yes

Are all factual statements correct and adequately supported by citations?

Yes

Are the conclusions drawn appropriate in the context of the current research literature?

Yes

Is the topic of the review discussed comprehensively in the context of the current literature?

Yes

Reviewer Expertise:

My areas of research include artificial intelligence (AI) and its applications in healthcare, ethical frameworks for AI, data privacy and security, and the intersection of technology with societal and cultural contexts. I also focus on leveraging AI for sustainable development, exploring interdisciplinary approaches to address global challenges, and enhancing data-driven decision-making in various sectors.

I confirm that I have read this submission and believe that I have an appropriate level of expertise to confirm that it is of an acceptable scientific standard.

# Reviewer report: Reviewer response for version 1

**DOI:** 10.21956/wellcomeopenres.25352.r108619

**Published:** 2024-11-25

**Authors:** Ali Husnain

**Affiliations:** 1 Chicago State University, Chicago, USA

The article titled *"The Ubuntu Way: Ensuring Ethical AI Integration in Health Research"* explores the ethical challenges and implications of artificial intelligence (AI) in health research, particularly in African contexts. It critiques the limitations of existing global AI ethics frameworks, which are predominantly shaped by Western philosophies emphasizing individualism. To address these gaps, the authors propose a framework based on Ubuntu, an African philosophy that values communalism, interconnectedness, and collective well-being.

 The paper highlights four key principles derived from Ubuntu:

1. **Communalism and Openness** – Emphasizing the collective well-being of communities.
2. **Harmony and Support** – Promoting mutual support and inclusion.
3. **Research Prioritization and Community Empowerment** – Ensuring equitable access and long-term sustainability.
4. **Community-Oriented Decision-Making** – Involving communities in AI development and implementation processes.

These principles are aligned with global ethical standards, such as justice, beneficence, and sustainability, but are adapted to reflect African cultural values. The article concludes that integrating Ubuntu into AI health research ethics can create more contextually relevant and equitable frameworks, not just for Africa but potentially on a global scale.

 Evaluation of the Article

 1. **Is the topic of the review discussed comprehensively in the context of the current literature?**

- **Answer:** Yes.
- **Comments:**
  - The article provides a robust discussion of the gaps in current AI ethics frameworks and contrasts them with Ubuntu-based principles.
  - It incorporates a broad range of sources, including policy documents, peer-reviewed articles, and practical examples from African countries (e.g., Nigeria, Kenya, Zambia). This contextual grounding strengthens the paper.
  - The authors engage critically with the literature, identifying ethical issues at individual, community, national, and environmental levels, and positioning Ubuntu as a culturally relevant solution.

2. **Are all factual statements correct and adequately supported by citations?**

- **Answer:** Yes.
- **Comments:**
  - All factual claims, including those about AI applications in Africa and existing ethics frameworks, are supported by credible sources.
  - The examples, such as Ubenwa's AI diagnosis of birth asphyxia and AI-driven drug identification in Nigeria, are well-documented and enhance the paper’s credibility.

3. **Is the review written in accessible language?**

- **Answer:** Yes.
- **Comments:**
  - The paper is well-written and accessible to a broad audience, including those unfamiliar with AI ethics or African philosophy.
  - Complex concepts like Ubuntu and AI methodologies are clearly explained without excessive jargon.

4. **Are the conclusions drawn appropriate in the context of the current research literature?**

- **Answer:** Yes.
- **Comments:**
  - The conclusions are well-grounded in the evidence and align with the identified gaps in the literature.
  - The proposed framework is a logical extension of the discussion and offers practical and theoretical contributions to AI ethics in health research.

Areas for Improvement

 1. **Global Relevance of Ubuntu**

- **Issue:** The paper primarily focuses on African contexts but does not fully address how Ubuntu principles could inform global AI ethics frameworks.
- **Recommendation:** Include a section discussing how Ubuntu’s emphasis on communalism, environmental stewardship, and inclusivity might address global challenges, such as algorithmic bias and environmental sustainability. Highlighting this would broaden the paper’s appeal and impact.

2. **Clarifying "Alignment" with Global Frameworks**

- **Issue:** The paper frequently states that Ubuntu "aligns" with international ethical principles but does not elaborate on how it might transcend or enhance them.
- **Recommendation:** Expand the discussion to emphasize how Ubuntu provides a more integrative and relational framework compared to the often fragmented principles of Western ethics. For example, highlight how Ubuntu’s focus on collective well-being offers a more cohesive approach to justice, beneficence, and sustainability.

3. **Operationalizing Ubuntu in Practice**

- **Issue:** While the paper proposes an Ubuntu-based framework, it lacks detailed guidance on how this framework can be implemented in real-world health research projects.
- **Recommendation:** Include practical examples or case studies illustrating how Ubuntu principles could be applied in AI-driven health research. For instance, describe how community consent processes or environmental impact assessments might look under an Ubuntu framework.

4. **Addressing Limitations**

- **Issue:** The limitations of Ubuntu, such as its potential misappropriation or ambiguity in defining “community,” are briefly mentioned but not explored in depth.
- **Recommendation:** Provide more detailed analysis of these limitations and suggest strategies to address them. For example:
  - How can researchers ensure Ubuntu is not superficially adopted by non-African researchers?
  - How should the concept of "community" be operationalized to account for diverse and overlapping identities (local, national, and global)?

Required Revisions for Scientific Soundness

 To ensure the article is scientifically sound and impactful, the following points should be addressed:

1. **Expand Global Applicability:** Discuss how Ubuntu principles could inform global AI ethics, particularly in addressing issues like algorithmic bias and environmental sustainability.
2. **Enhance Practical Guidance:** Provide specific examples or case studies illustrating how the proposed framework can be implemented in AI health research projects.
3. **Clarify Alignment and Distinction:** Elaborate on how Ubuntu goes beyond aligning with international frameworks to offer a more cohesive ethical approach.
4. **Deepen Discussion of Limitations:** Explore the practical and theoretical challenges of applying Ubuntu, particularly in multicultural or global contexts.

Overall Assessment

 This is a well-researched and timely paper that offers a unique perspective on AI ethics in health research. By incorporating Ubuntu, the authors address critical gaps in existing frameworks and propose a culturally relevant and holistic approach to ethical AI governance. With the suggested revisions, this work has the potential to make a significant contribution to the field of AI ethics, both in Africa and globally.

Is the review written in accessible language?

Yes

Are all factual statements correct and adequately supported by citations?

Yes

Are the conclusions drawn appropriate in the context of the current research literature?

Yes

Is the topic of the review discussed comprehensively in the context of the current literature?

Yes

Reviewer Expertise:

AI ethics, healthcare research, bioethics, African philosophy, data governance, and public health technology.

I confirm that I have read this submission and believe that I have an appropriate level of expertise to confirm that it is of an acceptable scientific standard, however I have significant reservations, as outlined above.

# Reviewer report: Reviewer response for version 1

**DOI:** 10.21956/wellcomeopenres.25352.r109768

**Published:** 2024-11-16

**Authors:** James Andrew Shaw

**Affiliations:** 1 University of Toronto, Toronto, Ontario, Canada

Thank you for the opportunity to review this excellent paper providing a summary of the philosophical concept of Ubuntu, its implications for AI health research, and its relation to Western and international ethical ideals that have been used to frame AI health research guidance internationally. I have just two minor comments on this paper that the authors may choose to address.

 First, after reading the paper, I am left wondering how researchers outside of the African context might best engage with the content. The authors clearly state that their aim is to focus on “the importance of embedding African philosophies within African countries’ AI health research ethics frameworks”. This is entirely appropriate and sufficient for the paper, and in this way the paper is really intended for an African audience (which is excellent). My reflection comes from a recognition that Ubuntu offers a coherence across ethical principles that seems absent in ethical conversations driven by Western perspectives, thereby offering something of immense value to Western and global discussions about the ethics of AI health research. Do the authors believe that there are one or two central insights that authors outside of African contexts should carry forward after learning about Ubuntu? Or, in the spirit of decolonizing global health, is this simply not the intent of the paper? Would the authors instead caution against authors in Western contexts drawing on Ubuntu out of risk that it is done in a superficial, colonial way?

 Second, and related, the authors describe how Ubuntu “aligns” with principles or practices of international ethics frameworks. Having read this section, my sense is that Ubuntu not only “aligns” with these perspectives, but also offers a convergence between those diverse ethical ideals that cohere on the notion of community good. In this way, Ubuntu offers a more powerful notion than the otherwise disconnected ideals of Western ethics, because it offers an integrating framework across these principles and practices. I would suggest that the authors state this in their introductory paragraph to the section comparing Ubuntu to Western ethical perspectives so as to more clearly emphasize the unique contributions of Ubuntu.

Is the review written in accessible language?

Yes

Are all factual statements correct and adequately supported by citations?

Yes

Are the conclusions drawn appropriate in the context of the current research literature?

Yes

Is the topic of the review discussed comprehensively in the context of the current literature?

Yes

Reviewer Expertise:

Bioethics, AI ethics, health policy

I confirm that I have read this submission and believe that I have an appropriate level of expertise to confirm that it is of an acceptable scientific standard.
